# Deep Learning in Medical Image Analysis

**DOI:** 10.3390/jimaging7040074

**Published:** 2021-04-20

**Authors:** Yudong Zhang, Juan Manuel Gorriz, Zhengchao Dong

**Affiliations:** 1 School of Informatics, University of Leicester, Leicester LE1 7RH, UK; 2 Department of Signal Theory, Telematics and Communications, University of Granada, 18071 Granada, Spain; gorriz@ugr.es; 3 Molecular Imaging and Neuropathology Division, Columbia University and New York State Psychiatric Institute, New York, NY 10032, USA; zhengchao.dong@nyspi.columbia.edu

Over recent years, deep learning (DL) has established itself as a powerful tool across a broad spectrum of domains in imaging—e.g., classification, prediction, detection, segmentation, diagnosis, interpretation, reconstruction, etc. While deep neural networks were initially nurtured in the computer vision community, they have quickly spread over medical imaging applications.

The accelerating power of DL in diagnosing diseases will empower physicians and speed-up decision making in clinical environments. Applications of modern medical instruments and digitalization of medical care have led to enormous amounts of medical images being generated in recent years. In the big data arena, new DL methods and computational models for efficient data processing, analysis, and modeling of the generated data are crucial for clinical applications and understanding the underlying biological process.

The purpose of this Special Issue (SI) “Deep Learning in Medical Image Analysis” is to present and highlight novel algorithms, architectures, techniques, and applications of DL for medical image analysis.

This SI called for papers in April 2020. It received more than 60 submissions from over 30 different countries. After strict peer reviews, only 22 papers were accepted and published. A total of 18 papers are research articles and the remaining 4 are review papers.

Leuschner and Schmidt (2021) [1] from Germany, the Netherlands, and Canada present the results of a data challenge that the authors organized, bringing together algorithm experts from different institutes to jointly work on quantitative evaluation of several data-driven methods on two large, public datasets during a ten-day sprint.

Shirokikh and Shevtsov (2021) [2] from Russia propose a new segmentation method with a human-like technique to segment a 3D study. Their method not only reduces the inference time from 10min to 15s, but also preserves state-of-the-art segmentation quality.

Zhang and Li (2021) [3] from China and the USA propose a meta-learning algorithm to augment the existing algorithms with the capability to learn from diverse segmentation tasks across the entire task distribution. The authors conduct experiments using a diverse set of segmentation tasks from the Medical Segmentation Decathlon and two meta-learning benchmarks.

Nannavecchia and Girardi (2021) [4] from Italy present a system able to automatically detect the causes of cardiac pathologies in electrocardiogram (ECG) signals from personal monitoring devices, with the aim to alert the patient to send the ECG to the medical specialist for a correct diagnosis and proper therapy.

Furtado (2021) [5] from Portugal takes on three different medical image segmentation problems: (i) segmentation of organs in magnetic resonance images, (ii) liver in computer tomography images, and (iii) diabetic retinopathy lesions in eye fundus images. The author quantifies loss functions and variations, as well as segmentation scores of different targets. The author concludes that dice is the best.

Shimizu and Hachiuma (2021) [6] from Japan combine three modules for localization, selection, and classification for the detection of the two surgical tools. In the localization module, the authors employ the Faster R-CNN to detect surgical tools and target hands, and in the classification module, the authors extract hand movement information by combining ResNet-18 and long short-term memory (LSTM) to classify two tools.

Bourouis and Alharbi (2021) [7] from Saudi Arabia and Canada introduce a new statistical framework to discriminate patients who are either negative or positive for certain kinds of virus and pneumonia. The authors tackle the current problem via a fully Bayesian approach based on a flexible statistical model named shifted-scaled Dirichlet mixture models.

Andrade and Teixeira (2021) [8] from Portugal present a technique to efficiently utilize the sizable number of dermoscopic images to improve the segmentation capacity of macroscopic skin lesion images. The quantitative segmentation results are demonstrated on the available macroscopic segmentation databases, SMARTSKINS and Dermofit Image Library.

Kandel and Castelli (2020) [9] from Portugal and Slovenia study an appropriate method to classify musculoskeletal images by transfer learning and by training from scratch. The authors apply six state-of-the-art architectures and compare their performances with transfer learning and with a network trained from scratch.

Comelli (2020) [10] from Italy presents an algorithm capable of achieving the volume reconstruction directly in 3D by leveraging an active surface algorithm. The results confirm that the active surface algorithm is superior to the active contour algorithm, outperforming an earlier approach on all the investigated anatomical districts with a dice similarity coefficient of 90.47 ± 2.36% for lung cancer, 88.30 ± 2.89% for head and neck cancer, and 90.29 ± 2.52% for brain cancer.

The methodology proposed by Ortega-Ruiz and Karabağ (2020) [11] from Mexico and the United Kingdom is based on traditional computer vision methods (K-means, watershed segmentation, Otsu’s binarization, and morphological operations), implementing color separation, segmentation, and feature extraction. The methodology is validated with the score assigned by two pathologists through the intraclass correlation coefficient.

The main aim of Kandel and Castelli (2020) [12] from Portugal and Slovenia is to improve the robustness of the classifier used by comparing six different first-order stochastic gradient-based optimizers to select the best for this particular dataset. Their results show that the adaptative-based optimizers achieved the highest results, except for AdaGrad, which achieved the lowest results.

La Barbera and Polónia (2020) [13] from Italy and Portugal employ a pipeline based on a cascade of deep neural network classifiers and multi-instance learning to detect the presence of HER2 from haematoxylin–eosin slides, which partly mimics the pathologist’s behavior by first recognizing cancer and then evaluating HER2.

Khoshdel and Asefi (2020) [14] from Canada employ a 3D convolutional neural network, based on the U-Net architecture, that takes in 3D images obtained using the contrast-source inversion method and attempts to produce the true 3D image of the permittivity.

Dupont and Kalinicheva (2020) [15] from France proposes a DL architecture that can detect changes in the eye fundus images and assess the progression of the disease. Their method is based on joint autoencoders and is fully unsupervised. Their algorithm has been applied to pairs of images from time series of different eye fundus images of 24 age-related macular degeneration patients.

Almeida and Santos (2020) [16] from Brazil propose a strategy for the analysis of skin images, aiming to choose the best mathematical classifier model for the identification of melanoma, with the objective of assisting the dermatologist in the identification of melanomas, especially towards an early diagnosis.

Tang and Kumar (2020) [17] from the USA propose a deep multimodal model that learns a joint representation from two types of connectomic data offered by fMRI scans. Their multimodal training strategy achieves a classification accuracy of 74% and a recall of 95%, as well as an F1 score of 0.805, and its overall performance is superior to that of using only one type of functional data.

In the work of Pintelas and Liaskos (2020) [18] from Greece, an accurate and interpretable machine learning framework is proposed for image classification problems able to make high quality explanations. Their results demonstrate the efficiency of the proposed model since it managed to achieve sufficient prediction accuracy, which is also interpretable and explainable in simple human terms.

Kieu and Bade (2020) [19] from Malaysia and the United Kingdom present a taxonomy of the state-of-the-art DL-based lung disease detection systems, visualize the trends of recent work on the domain and identify the remaining issues and potential future directions in this domain.

In the survey of Debelee and Kebede (2020) [20] from Ethiopia and Germany, several DL-based approaches applied to breast cancer, cervical cancer, brain tumor, colon and lung cancers are studied and reviewed. The result of the review process indicates that DL methods are the state-of-the-art in tumor detection, segmentation, feature extraction and classification.

Aruleba and Obaido (2020) [21] from South Africa provide a concise overview of past and present conventional diagnostics approaches in breast cancer detection. Further, the authors give an account of several computational models (machine learning, deep learning, and robotics), which have been developed and can serve as alternative techniques for breast cancer diagnostics imaging.

Singh and Sengupta (2020) [22] from Canada present a review of the current applications of explainable deep learning for different medical imaging tasks. The various approaches, challenges for clinical deployment, and the areas requiring further research are discussed in this review from a practical standpoint of a deep learning researcher designing a system for the clinical end-users.

The 22 accepted papers in this SI are from 19 countries: Brazil, Canada, China, Ethiopia, France, Germany, Greece, Italy, Japan, Malaysia, Mexico, Netherlands, Portugal, Russia, Saudi Arabia, Slovenia, South Africa, the UK, and the USA.

All the three Guest Editors hope that this Special Issue “Deep Learning in Medical Image Analysis” will benefit the scientific community and contribute to the knowledge base, and would like to take this opportunity to applaud the contributions of all the authors in this Special Issue. The contributions and efforts of the reviewers to enhance the quality of the manuscripts are also much appreciated. It is also necessary to acknowledge the assistance provided by the MDPI editorial team who make our GE tasks much easier.
